# Editorial: The Impact of Dietary Changes on Non-communicable Diseases in Latin America

**DOI:** 10.3389/fnut.2022.881676

**Published:** 2022-03-28

**Authors:** Marcia Cristina Teixeira Martins, Joan Sabaté

**Affiliations:** ^1^Center for Health Sciences Research, School of Medicine and Health Sciences, Adventist University of Plata, Libertador San Martin, Argentina; ^2^Center for Nutrition, Lifestyle, and Disease Prevention, School of Public Health, Loma Linda University, Loma Linda, CA, United States

**Keywords:** dietary changes, non-communicable disease, Latin America, plant-based foods, food choices

It is estimated that the four major non-communicable diseases (NCDs)—cardiovascular disease, most cancers, diabetes, and chronic respiratory diseases—will account for ~81% of deaths in Latin America and the Caribbean by 2030 (1). According to the report of the Global Burden of Disease (2), dietary risks (e.g., low intake of unprocessed plant foods and high sodium consumption) contributed to the rise of burden from chronic diseases in Latin America and to DALY (disability-adjusted life years) worldwide. Likewise, recent reports show that total intake of dairy, fish, vegetables, fruits, legumes, whole grains, nuts, and seeds were below reference dietary intake (3) in Latin America. In fact, diet is the leading risk factor for premature death and disability in the region. However, and despite this fact, there is a lack of knowledge relating to the link between diet and major aspects of the epidemic of NCDs that are prevalent in this region, such as: factors impacting food choices and dietary changes (e.g., COVID-19 pandemic), evidence from diet and lifestyle advice, eating habits, vulnerable groups, beneficial foods and food patterns (e.g., plant-based, Mediterranean), among others. Therefore, our Research Topic is entitled: “*The Impact of Dietary Changes on Non-Communicable Diseases in Latin America*” so as to attract investigators from different Latin American countries in order to improve our understanding of the complexity of dietary risks on NCDs.

Ten papers were accepted after peer revision, including data from five Latin American countries (Argentina, Brasil, Chile, Mexico, and Peru): six cross-sectional studies (three descriptive, two population-based and one mixed-method), one tool development research article, and three reviews.

The review paper of Matos et al. assesses current trends in Latin America with respect to consumption of ultra-processed foods (UPF) which, together with other social and environmental factors, influences the prevalence of NCDs. An overview of UPS, concerns and trends in the intake, social determinants in the sales, the link between the intake of UPS and NCDs, as well as potential mechanisms are discussed. Mexico and Chile seem to be consuming the most UPF per capita.

In line with this finding, Araya et al. evaluated 960 pre-scholars from Santiago (Chile) and observed that UPF is the primary source of energy intake (49% of the total energy). Children in the highest quintile of UPF intake show higher levels of energy, saturated and monounsaturated fats, carbohydrates, total sugars and vitamin D compared to those in the lowest quintile. Conversely, there is an inverse association of UPF with consumption of proteins, polyunsaturated fats, fiber, zinc, vitamin A, and sodium.

Considering also the increase in the occurence of NCDs with advancing age, Loureiro et al. performed a population-based cross-sectional study with a sample of 621 community-dwelling older adults from Viçosa, Minas Gerais, Brazil. The authors constructed their food consumption profiles (“unhealthy,” “less unhealthy,” and “fairly healthy”) from food frequency questionnaire data utilizing a two-step cluster method. They found that illiterate or semi-literate individuals, those with low income, and those who neglect to seek medical advice require greater attention and care concerning dietary habits. Therefore, these groups should be prioritized when formulating healthy eating actions and programs aiming prevention and control of NCDs.

In a cross sectional study of a Northeast Brazilian rural vulnerable community of African-descendants (*quilombola*) and *non-quilombola* adolescentes, Cairo et al. finds no difference in overweight between the two groups. Overweight is directly associated with screen time and inversely associated with breakfast consumption and school attendance, highlighting the importance of adopting a healthy lifestyle early in life and the role of school for health promotion in rural regions.

In fact, the lifestyle medicine (LM) approach includes dietary recommendations coupled with other lifestyle practices (also known as LM pillars) for the prevention and treatment of NCDs. Two papers of our Research Topic address this issue. Figueroa et al. highlight the plant-based Mediterranean diet as a LM-contextualized dietary pattern that is adaptable, feasible, and sustainable within the Chilean context with potential health impact for handling the prevalence of risk factors and NCDs in this country. Urbano et al. assessed the reception of diet and other health-related lifestyle advice (HRA) to address NCDs in a primary care context in Central Argentina. Although tailored, written, and detailed recommendations were highly appreciated by patients, it was found that such advice is still underutilized and mostly offered in the context of illness. The study emphasizes the value of using this simple tool to help address patient's needs to prevent and control NCDs in Argentina.

Low cost fermented probiotic kefir is presented in the Peluzio et al. narrative review as a potential strategy for the prevention of microbiota-related metabolic NCDs in Latin America and other parts of the world. For that purpose, it would be necessary to focus on the standardization of production protocols with careful selection of microorganisms to be present in the starter culture and the final drink.

One of the reasons for a lack of studies relating dietary-associated risk factors for NCDs in Latin American countries is the need for adequate instruments and tools to assess food consumption. For this reason, Contreras-Guillén et al. developed an open-access automated tool (MAR_24_) for collecting 24-h dietary recalls using the United States Department of Agriculture (USDA) Automated Multiple-Pass Method (AMPM).

Latin American countries are also in need of adequate references for anthropometric variables to evaluate health, dietary status, disease risks (including NCD risk), and changes in body composition. Therefore, we included a study from Gómez-Campos et al. conducted in 15,436 children, youths and adults in the Maule region of Chile. The authors compare anthropometric indicators with American references and propose percentiles for evaluating nutritional status of the Chilean population between 5 and 80 years old.

The manuscript submission period for this Research Topic took place during the first wave of the COVID-19 pandemic which severely affected eating and lifestyle habits and threatened patients with NCDs in Latin America and worldwide. For this reason, the cross-sectional investigation of Enriquez-Martinez et al. is included, showing diet and lifestyle changes during the first wave of the pandemic in five Ibero-American countries. We hope that their findings can be used to guide policies to prevent consequences that may affect the incidence of chronic diseases.

All the studies included in this Research Topic either directly or indirectly explore the link between diet and NCDs in Latin America. Finally, we thank *Frontiers in Nutrition* for the opportunity to contribute to this important topic. We acknowledge our valued authors who, together with the manuscript reviewers, joined efforts to bring relevant information on the relationship between diet and chronic diseases in Latin America.

## Author Contributions

MCTM drafted the manuscript. JS reviewed and edited the manuscript. Both authors contributed to the article and approved the submitted version.

## Conflict of Interest

The authors declare that the research was conducted in the absence of any commercial or financial relationships that could be construed as a potential conflict of interest.

## Publisher's Note

All claims expressed in this article are solely those of the authors and do not necessarily represent those of their affiliated organizations, or those of the publisher, the editors and the reviewers. Any product that may be evaluated in this article, or claim that may be made by its manufacturer, is not guaranteed or endorsed by the publisher.
